# Combined TIM-3 blockade and CD137 activation affords the long-term protection in a murine model of ovarian cancer

**DOI:** 10.1186/1479-5876-11-215

**Published:** 2013-09-17

**Authors:** Zhiqiang Guo, Dali Cheng, Zhijun Xia, Meng Luan, Liangliang Wu, Gang Wang, Shulan Zhang

**Affiliations:** 1 Department of Gynecology and Obstetrics, Shengjing Hospital, China Medical University, ShenYang 110004, China; 2 Department of Gynecology and Obstetrics, The First Affiliated Hospital, China Medical University, Shen Yang 110001, China; 3 Cancer Center Key Lab, PLA General Hospital, Beijing 100853, China

## Abstract

**Background:**

T-cell immunoglobulin and mucin domain 3 (TIM-3) is known as a negative immune regulator and emerging data have implicated TIM-3 a pivotal role in suppressing antitumor immunity. The co-stimulatory receptor CD137 is transiently upregulated on T-cells following activation and increases their proliferation and survival when engaged. Although antagonistic anti-TIM-3 or agonistic anti-CD137 antibodies can promote the rejection of several murine tumors, some poorly immunogenic tumors were refractory to this treatment. In this study, we sought to evaluate whether combined TIM-3 blockade and CD137 activation would significantly improve the immunotherapy in the murine ID8 ovarian cancer model.

**Methods:**

Mice with established ID8 tumor were intraperitoneally injected with single or combined anti-TIM-3/CD137 monoclonal antibody (mAb); mice survival was recorded, the composition and gene expression of tumor-infiltrating immune cells in these mice was analyzed by flow cytometry and quantitative RT-PCR respectively, and the function of CD8^+^ cells was evaluated by ELISA and cytotoxicity assay.

**Results:**

Either anti-TIM-3 or CD137 mAb alone, although effective in 3 days established tumor, was unable to prevent tumor progression in mice bearing 10 days established tumor, however, combined anti-TIM-3/CD137 mAb significantly inhibited the growth of these tumors with 60% of mice tumor free 90 days after tumor inoculation. Therapeutic efficacy was associated with a systemic immune response with memory and antigen specificity, required CD4^+^ cells and CD8^+^ cells. The 2 mAb combination increased CD4^+^ and CD8^+^ cells and decreased immunosuppressive CD4^+^FoxP3^+^ regulatory T (Treg) cells and CD11b^+^Gr-1^+^ myeloid suppressor cells (MDSC) at tumor sites, giving rise to significantly elevated ratios of CD4^+^ and CD8^+^ cells to Treg and MDSC; This is consistent with biasing local immune response towards an immunostimulatory Th1 type and is further supported by quantitative RT-PCR data showing the increased Th1-associated genes by anti-TIM-3/CD137 treatment. The increased CD8^+^ T cells produced high level of IFN-γ upon tumor antigen stimulation and displayed antigen-specific cytotoxic activity.

**Conclusions:**

To our knowledge, this is the first report investigating the effects of anti-TIM-3/CD137 combined mAb in a murine ovarian cancer model, and our results may aid the design of future trials for ovarian cancer immunotherapy.

## Background

Epithelial ovarian carcinoma (EOC) is the leading cause of death from gynecologic malignancies in the United States and is the fourth most common cause of cancer death in women [1]. Over 70% of women with EOC present with advanced stage disease and tumor dissemination throughout the peritoneal cavity [2]. Despite the standard therapy with surgical cytoreduction and the combination of cisplatin and paclitaxel, the treatment efficacy is significantly limited by the frequent development of drug resistance [3]. Novel complementary strategies are urgently needed to improve the outcomes of ovarian cancer.

Much data suggest that immunotherapy for EOC should be effective [4]. Firstly, EOC cells express tumor-associated antigens against which specific immune responses have been detected [5-9]. Secondly, the studies pioneered by Coukos and colleagues indicate tumor immune surveillance plays a role in clinical outcomes in EOC supported by the close correlation between survival and tumor infiltration with CD3^+^ T cells in the large annotated clinical samples [10]. Thirdly, although EOC is a devastating disease, metastases are frequently restricted to the peritoneal cavity where the tumor microenvironment is directly accessible, which prevents the need for systemic delivery of immunostimulatory treatments [11]. Despite the abundant evidence that anti-tumor immunity could be effective, clinical success with immune-based therapies for EOC has generally been modest [12].

T-cell immunoglobulin and mucin domain 3 (TIM-3), as a relatively newly described co-inhibitory molecule, was expressed by IFN-γ–secreting T-helper 1 (Th1) cells and subsequently on CD8^+^ T cytotoxic type 1 (Tc1) cells, DCs and monocytes [13-16]. The galectin-9, a soluble molecule widely expressed and upregulated by IFN-γ, was identified as TIM-3 ligand [17,18], which induces cell death via binding to TIM-3 expressed on Th1 cells [19], suggesting a role for TIM-3 in negatively regulating Th1 responses. Emerging data has implicated TIM-3 a critical role in regulating tumor immune response. Early studies reported that the growth of 4 T1 mammary tumors was inhibited in TIM-3-deficient mice, and anti-TIM-3 monoclonal antibody (mAb) could suppress the growth of established subcutaneous EL4 lymphoma, suggesting TIM-3 as a potential target for cancer immunotherapy [20]. Recent studies observed that the expression of TIM-3 and PD-1 was up-regulated on circulating tumor-specific and tumor-infiltrating CD8^+^ T cells from patients and mice bearing advanced malignancies respectively, which correlated with the severely exhausted phenotype defined by failure to proliferate and produce effector cytokines, and combined blockade of both TIM-3 and PD-1 pathway reversed tumor-induced T-cell dysfunction and effectively suppressed the experimental tumor growth. This finding is further validated by the experiments demonstrating that combined anti-TIM-3/PD-1 mAbs significantly prevented established tumor growth and even cured a fraction of mice in methylcholanthrene-induced fibrosarcomas and 6 different experimental mouse tumor models, supporting the potential of blocking TIM-3 in combination with other immune-regulatory mAbs for the treatment of cancer.

CD137 (as known as CD137) belongs to the Tumor Necrosis Factor Receptor (TNFR) superfamily and is transiently upregulated on both CD4^+^ and CD8^+^ T cells following activation [21]. Upon engagement, CD137 co-stimulates CD8^+^ T cells promoting their proliferation, Th1-type cytokine production, and survival [22]. Much evidence demonstrate the promising effects for anti-CD137 mAbs in the treatment of mice bearing established tumors [23,24]; this is not only achieved by agonist antibodies but also by dimeric RNA aptamers or tumor cells expressing a surface-attached anti-CD137 single chain antibody [25,26]. This preclinical evidence has led to clinical trials with 2 human mAbs directed against CD137 [27].

Although antagonist TIM-3 or agonistic CD137 antibodies can promote the rejection of some murine tumors, however, poorly immunogenic tumors such as ID8 ovarian cancer do not respond to antibody therapy alone [28]. We hypothesized that combined TIM-3 blockade and CD137 activation would strengthen the antitumor effect by synergistically releasing the brake for CD4^+^ cells and promoting the function of CD8^+^ cells. In this study, using ID8 murine ovarian cancer model, we evaluated the therapeutic effect of single or combined anti-TIM-3 and anti-CD137 mAbs and found that combined anti-TIM-3/CD137 significantly suppressed the 10 days established peritoneal ID8 tumor growth, resulting in 60% of treated mice tumor free 90 days after tumor injection. We further characterized the cellular and molecular mechanisms driving this combined antitumor effect elucidating the basic processes necessary to achieve immune-mediated tumor rejection.

## Methods

### Mice

Female C57BL (6–8 wk old) were purchased from the Animal Experimental Center of the China Medical University. Animal use was approved by our institution (China Medical University).

### Cell lines

ID8, a clone of the MOSEC ovarian carcinoma of C57BL/6 origin was a gift from Dr. George Coukos (University of Pennsylvania, Philadelphia, USA). Murine B16 melanoma cells, TC-1 lung carcinoma cells and T cell lymphoma EL4 cells were purchased from ATCC (Manassas, VA). Tumor cells were cultured in the complete DMEM medium supplemented with 10% FBS (Thermo Scientific, Rockford, IL), 100 U/mL penicillin and 100 μg/mL streptomycin before cell suspensions were prepared and transplanted to mice. The EL4 cells and splenocytes were maintained in a complete medium of RPMI-1640 supplemented with 10% FBS, 25 mM HEPES, 2 mM glutamine, 100 U/mL penicillin and 100 μg/mL streptomycin.

### Antibodies

Therapeutic anti-CD137 (Clone lob12.3; Catalog#BE0169), anti-TIM-3 (Clone RMT3-23; Catalog#BE0115), anti-CD4 (Clone GK1.5; Catalog#:BE0003-1), anti-CD8 (Clone 2.43; Catalog#:BE0061), anti-NK1.1 (Clone PK136; Catalog#:BE0036), anti-CD19 (Clone 1D3; Catalog#:BE0150) and control (Clone 2A3; Catalog#:BE0089) were purchased from BioXcell (West Lebanon, NH). Antibodies used for flow cytometry were purchased from Tianjing Sungene (Tianjing, China) and eBioscience (San Diego, CA).

### Tumor challenge and treatment experiments

In the experiments with ID8 ovarian tumor (Additional file 1: Figure S1), mice (5 or 10 mice/group) were injected intraperitoneally (i.p.) with 1 × 10^6^ ID8 cells in 0.1 mL of PBS. At day 3, 7 and 11 (3 days established tumor model) or days 10, 14 and 18 (10 days established tumor model) post-tumor injection, each mouse received the i.p. injection of 250 μg of control, anti-TIM-3, anti-CD137 or combined anti-TIM-3/CD137 mAb in 250 μL of PBS as shown in the figure legends. The mice were weighted twice weekly and checked daily for the clinical sign of swollen bellies indicative of ascites information and for the evidence of toxicity such as respiratory distress, mobility, weight loss, diarrhea, hunched posture, and failure to eat while histopathology was conducted on major organs (i.e., liver, kidney, intestines, lungs, and colon). Following institutional guidelines, mice were killed when they developed ascites and had a weight increase > 30%. The survival of each mouse was recorded and overall survival was calculated.

For assessing the development of immune memory, pooled (2 independent experiment) 9 long-term surviving mice (90 days after first tumor injection) from combined anti-TIM-3/CD137 therapy group or age-matched naïve mice (which served as control) were challenged i.p. or subcutaneously (s.c.) with 1 × 10^6^ ID8 cells or 1 × 10^6^ syngeneic but antigenically different TC1 cells. Three perpendicular diameters of s.c. tumors were measured every second day using a caliper and tumor volumes were calculated according to the formula: 1/2 × (length) × (width)^2^. Mice were sacrificed when they seemed moribund or their tumors reached 10 mm in diameter.

For depletion of immune cells, mice were injected i.p. with 500 μg of mAbs to CD8, CD4, NK1.1, or CD19, 1 day before and two days after tumor challenge, followed by injection of 250 μg every 5 days throughout the experiment. The efficacy of cell depletion was verified by staining peripheral blood leukocytes for specific subsets after depletion (data not shown).

### Evaluation of tumor-infiltrating immune cells (TIIC) in peritoneal lavages by flow cytometry

Mice which had been transplanted i.p. with ID8 cells were euthanized 7 days after they had been injected with the 2 mAb combination (or control) as in the therapy experiments. To obtain peritoneal immune cells, 3 ml PBS was injected into the peritoneal cavity of mice with ID8 tumors immediately after euthanasia, their belly was massaged and the fluid was removed, filtered through a 70 μM cell strainer (BD Biosciences), washed and immune cells were isolated by using a mouse lymphocyte isolation buffer (Cedarlane, Burlington, Ontario) following the manufacturer’s instruction.

For the staining of immune cells, above prepared immune cells were washed with FACS staining buffer and incubated with mouse Fc receptor binding inhibitor (eBioscience) for 10 minutes before staining with mAbs (Tianjing Sungene) against mouse CD45 (clone 30-F11), CD3 (clone 145-2C11), CD4 (clone GK1.5), CD8 (clone 53–6.7), CD19 (clone eBio1D3), CD11b (clone M1/70) and Gr-1 (clone RB6-8C5) for 30 minutes. For intracellular staining of FoxP3 (clone FJK-16 s; eBioscience), cells were fixed, permeabilized and stained following the instruction of Cytofix/Cytoperm kit (BD Bioscience). Flow cytometry was performed using FACSCalibur (BD Biosciences) and the data were analyzed using FlowJo software (Tree Star). All flow cytometry experiments were performed at least 3 times.

### Quantitative RT-PCR

Total cellular RNA was extracted using RNeasy Mini Kits (Qiagen, Hilden, GA) and reverse transcribed into cDNA using SuperScript III Reverse Transcriptase (Invitrogen). Expression for genes of interest was analyzed in cells of peritoneal lavage on day 7 after the third injection of mAb. The primers for all genes tested, including internal control GAPDH, were synthesized by Takara Inc., Dalian, China. Primer sequences were listed in Additional file 2: Table S1. Quantitative real-time PCR was performed via ABI PRISM 7500 Real-Time PCR Systerm (Applied Biosystems) with 1× SYBR Green Universal PCR Mastermix (Takara). Transcript levels were calculated according to the 2–ΔΔCt method, normalized to the expression of GAPDH, and expressed as fold change compared with control.

### Evaluation of antigen-specific CTL immune response

Isolated splenocytes from treated mice were cultured in the presence of 10 μg/mL H-2Db-restricted mesothelin-derived peptides (amino acid 406–414) or control HPV-E7-derived peptide (amino acid 49–57; all from GenScript, Nanjing, CA) for 3 days. IFN-γ in the supernatants was determined by Mouse IFN-γ Quantikine ELISA Kit (R&D systems, Minneapolis, MN).

For CTL assays, effector cells were obtained by coculturing 5 × 10^6^ splenocytes with 5 × 10^5^ UV-irradiated ID8 cells for 4 days. Peptide-pulsed EL4 target cells were generated by adding 10 μg/ml of peptide and incubating for 4 hours. CTL activity was measured using the CytoTox96 Non-Radioactive Cytotoxicity Assay kit (Promega, Madison, WI) following the manufacturer’s instructions. In brief, target cells were incubated with varying numbers of effector cells for about 4 hours, and supernatants were then analyzed for lactate dehydrogenase release. The results are expressed as percent specific lysis, calculated as (Experimental release-Spontaneous release/Total release-Spontaneous release) × 100. In some experiments, effector cells were incubated with anti-CD4 or CD8 antibody (10 μg/mL) for 2 hours before CTL assay.

### Antibody evaluation by flow cytometry

We detected the presence of mesothelin-specific antibodies using the method described previously [29]. Blood was obtained from 2 mAb treated long-term surviving mice (90 days after the tumor inoculation). The presence of mesothelin-specific antibodies was determined by staining the mouse ID8 ovarian cancer cells using serum from treated mice in a 1:200 dilution, followed by Phycoerythrin-conjugated anti-mouse IgG antibody (eBioscience) staining. Staining with sera from naïve mice was used as negative control. Analysis of cell staining was performed described above.

### ELISA

Mice injected i.p. with 1 × 10^6^ ID8 cells 10 day earlier were injected thrice at 4 days interval with 250 μg of control or anti-TIM-3/CD137 mAb. Seven days after the last mAb injection, pooled peritoneal lavage cells (1 × 10^6^/well) harvested from treated mice were stimulated in vitro with 50 ng/ml PMA and 1 μg/ml ionomycin for 6 hours prior to the analysis of IL-10 and IFN-γ production in the supernatants by ELISA according to the manual (R&D systems). The results were analyzed after normalization according to the T cell numbers.

### Statistics

Results were expressed as mean ± SEM. All statistical analyses were performed using GraphPad Prism 5. Student’s t test was used to compare the statistical difference between two groups and one-way ANOVA was used to compare three or more groups. Survival rates were analyzed using the Kaplan–Meier method and evaluated with the log-rank test with Bonferroni correction. Significant differences were accepted at p < 0.05.

## Results

### Synergistic antitumor effect of anti-TIM-3 /CD137 mAb

**Figure 1:**
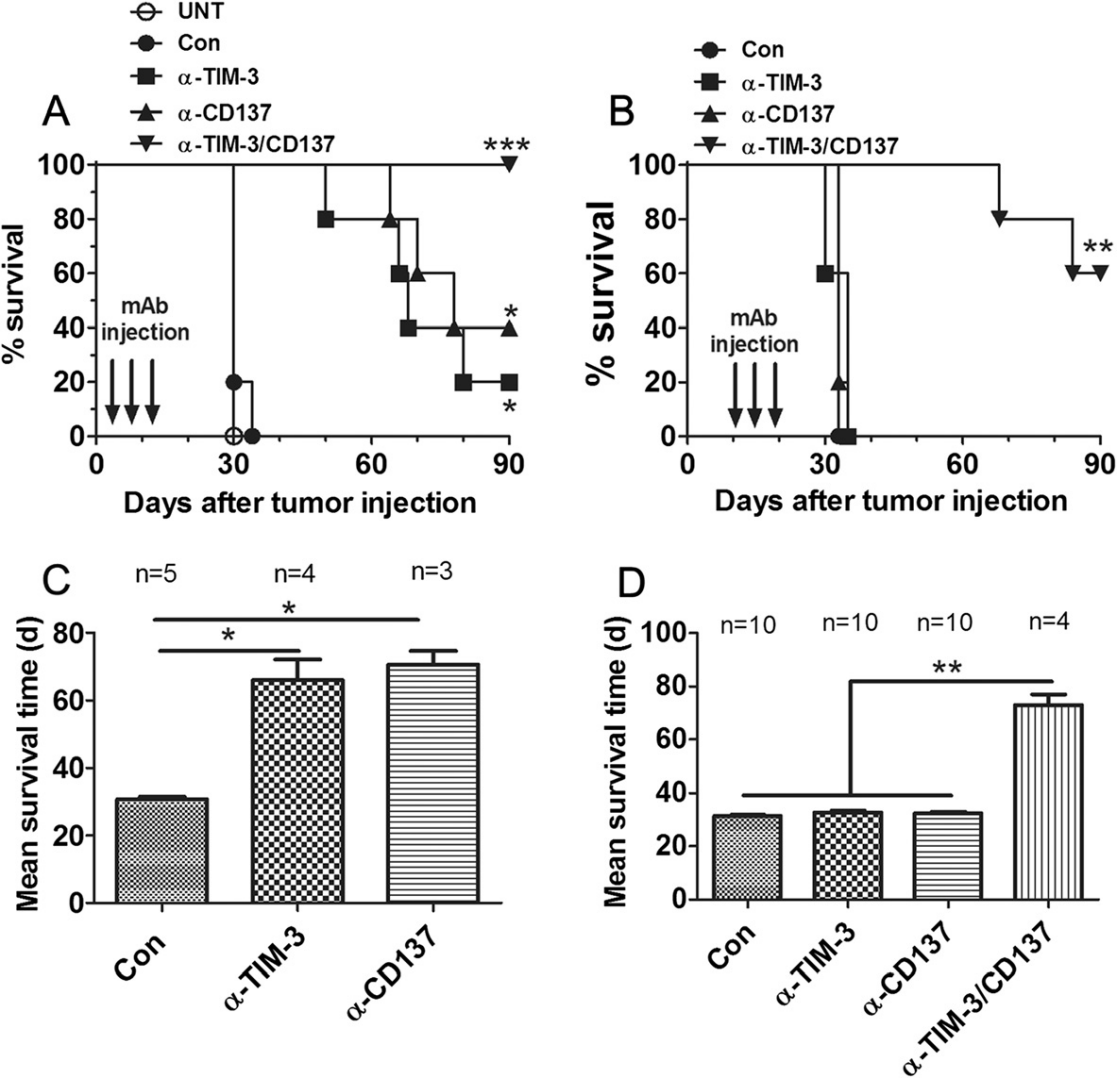
**Antitumor effects of individual or combined anti-TIM-3 and anti-CD137 mAbs in murine ID8 ovarian cancer model.** Mice (5 or 10 mice/group) transplanted i.p. with 1 × 10^6^ ID8 cells 3 **(A, C)** or 10 **(B, D)** day before were treated thrice with 250 μg of control, anti-TIM-3, anti-CD137 and anti-TIM-3/CD137 mAb at 4 days interval and overall survival of mice was recorded **(A, B)** and mean survival time of mice with tumor growth was calculated **(C, D)**. The experiment was repeated once with similar result. *P < 0.05, **P < 0.01, ***P < 0.001, compared with control mAb treated mice.

We tested the antitumor efficacy, defined as prolonged overall survival, of single or combined anti-TIM-3 and anti-CD137 mAb in C57BL/6 mice transplanted i.p. 3 or 10 days previously with 1 × 10^6^ ID8 cells. Untreated mice and mice receiving a control mAb developed ascites about 30 days post-injection and had to be euthanized (Additional file 1: Figure S1). As shown in Figure 1A, in 3 days established ID8 tumor model, single injection of three doses of anti-TIM-3 or anti-CD137 mAb significantly prolonged the survival of mice with 20% (1 out of 5 mice) or 40% (2 out of 5 mice) of mice surviving 90 days after tumor injection respectively when the experiment was terminated and euthanized mice were tumor free in peritoneal cavity, and even mice with tumor growth had increased mean survival time (MST) compared with control mAb treated mice (Figure 1C; MST 30.80, 66.00, 70.67 days for control, anti-TIM-3, anti-CD137 group; p < 0.05, anti-TIM-3 or anti-CD137 mAb compared to control mAb), however, using the same regimen, these two mAbs individually had little or no effects on the outgrowth of 10 days established ID8 tumor leading to ascites formation at the almost same time as control mAb treated mice. Intriguingly, combined treatment of anti-TIM-3 and anti-CD137 mAbs significantly increased survival of mice bearing 10 days established tumor with 60% (6 out of 10 mice) of mice tumor free 90 days after tumor injection (Figure 1B; p < 0.01, combined mAb compared to single or control mAb), and even mice succumbed to tumor growth also had significantly prolonged MTS compared with control or single mAb treated mice (Figure 1D; MTS 31.40, 32.80, 32.50 and 73.00 days for control, anti-TIM-3, anti-CD137 and anti-TIM-3/CD137 group; p < 0.01, combined mAb compared to single or control mAb). A repeat of the experiment gave similar results (data not shown). In addition, combined anti-TIM-3/CD137 mAbs were even more efficacious in 3 days established ID8 tumor model with 100% of mice remaining free of tumor 90 days post-injection (Figure 1A; p < 0.001 compared to control mAb). We did not detect any expression of TIM-3 and CD137 molecules and their respective ligands Galectin-9 and CD137L on the surface of ID8 ovarian cancer cells (data not shown), excluding the possibility that inhibition of ID8 tumor growth *in vivo* is directly mediated by anti-TIM-3 or anti-CD137 mAb.

**Figure 2:**
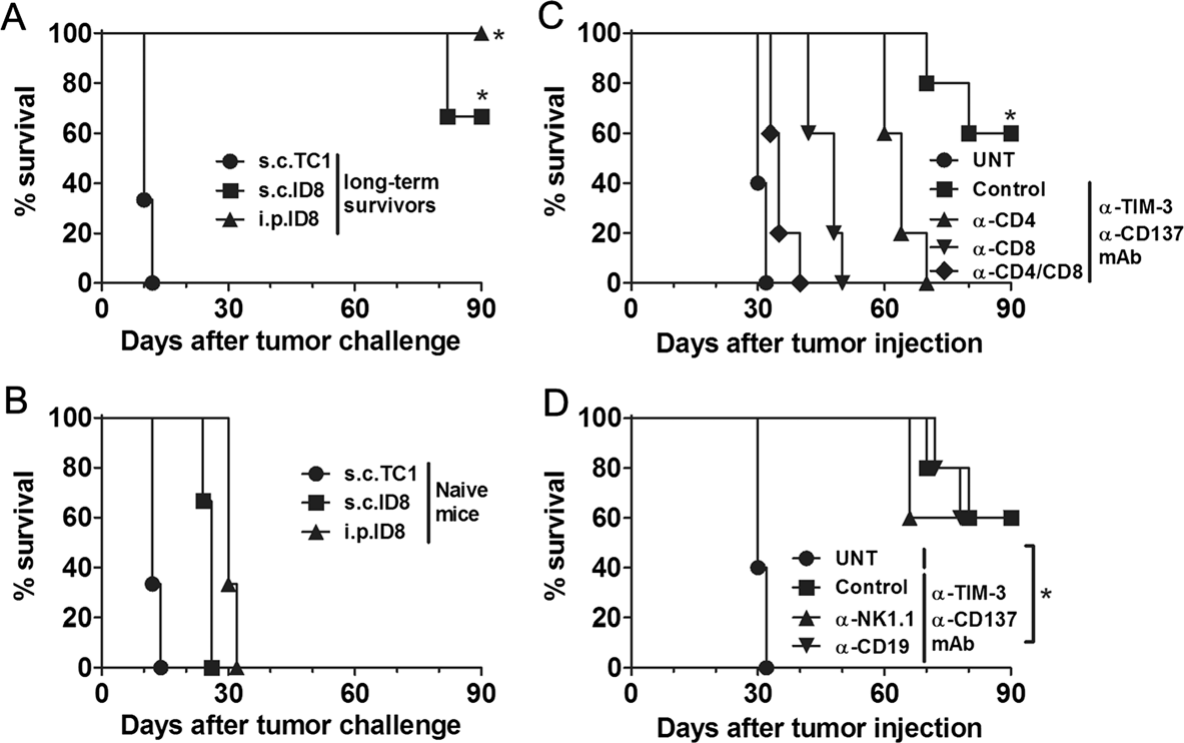
**Combined anti-TIM-3/CD137 mAb treatment induced tumor-specific long-lasting protection against ID8 ovarian cancer requiring CD4**^**+ **^**and CD8**^**+ **^**T cells.** The long-term survivors (90 days after first tumor challenge) pooled from 2 experiments were rechallenged (3 mice/group) with ID8 cells given i.p. or s.c. or with TC1 cells transplanted s.c. **(A)**; naive mice were transplanted with tumor cells as controls **(B)**. The survival of mice was recorded. **C and D**, Mice (5/group) treated with combined anti-TIM-3/CD137 mAb were also injected with an anti-CD4, anti-CD8, anti-CD4/CD8, anti-NK1.1, anti-CD19, or control mAb with 250 μg of each mAb per mouse 1 day before and two days after tumor challenge and every 5 days thereafter for the duration of the experiments. Tumor-bearing untreated mice were as negative controls (UNT). Data are representative of 2 experiments. *P < 0.05, s.c.TC1 compared with s.c.ID8 or i.p.ID8 in **A**; control depletion compared with CD4, CD8 or CD4/8 depletion in **C**; UNT compared with control, NK1.1 or CD19 depletion in **D**.

Notably, those long-term survivors developed the systemic tumor-specific memory immune response in that they were resistant to the rechallenge by both i.p. and s.c. injection of ID8 ovarian cancer cells but not s.c. injection of unrelated TC1 lung cancer cells (Figure 2A) while naïve mice succumbed to them (Figure 2B). Ninety days after rechallenge, 100% (3 out of 3 mice) or 66.7% (2 out of 3 mice) of mice remained tumor-free when rechallenged with i.p. or s.c. ID8 cells respectively. Antibody-mediated cell depletion experiments demonstrated that protection conferred by anti-TIM-3/CD137 mAbs was dependent on the CD4^+^ and CD8^+^ T cells as anti-TIM-3/CD137 mAbs was largely ineffective in the absence of CD4^+^ or CD8^+^ T cells (Figure 2C), but effective in the absence of mature B cells or NK cells (Figure 2D).

### Combined anti-TIM-3/CD137 mAb treatment strongly increases ratios of both CD8 and CD4 T cells to Treg and MDSC in peritoneal lavage

**Figure 3:**
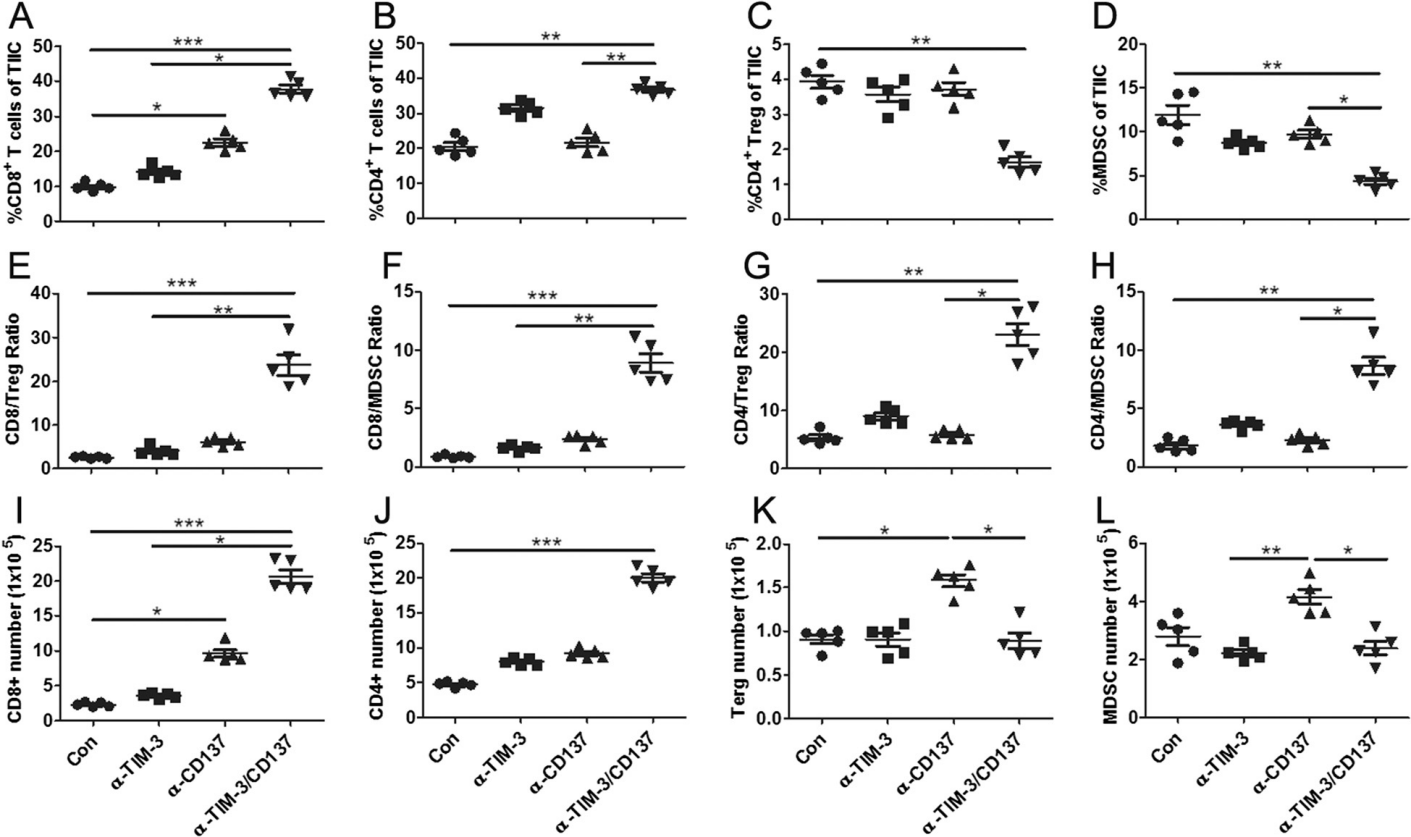
**Analysis of tumor-infiltrating immune cells (TIIC) in peritoneal lavage from treated mice.** Mice (5/group) injected i.p. with 1 × 10^6^ ID8 cells 10 day earlier were injected thrice at 4 days interval with 250 μg of control or anti-TIM-3/CD137 mAb. Seven days later, peritoneal lavage from treated mice was analyzed by flow cytometry for the percentages and numbers of various subsets of TIIC. The percentages of CD8^+^, CD4^+^, CD4^+^FoxP3^+^ Treg and CD11b^+^GR-1^+^ MDSC in peritoneal lavage are shown in **A, B, C and D** respectively with each dot representing data from each mouse. The ratios of CD8^+^ and CD4^+^ T cells to Treg and MDSC in peritoneal lavage are shown in **E, F, G and H** respectively with each dot representing data from each mouse. The absolute numbers of CD8^+^, CD4^+^, Treg and MDSC in peritoneal lavage are shown in **I, J, K and L** respectively with each dot representing data from each mouse. Data are representative of 2 independent experiments. *P < 0.05, **P < 0.01, ***P < 0.001.

To understand the apparent synergy between TIM-3 blockade and CD137 activation in the ID8 tumor model, we sought to dissect the effects of single or combined mAb on tumor-infiltrating immune cells (TIIC) in peritoneal lavage. Single CD137 engagement promoted the prominent infiltration of CD8 T cells in peritoneal cavity, but unchanged the relative fraction of CD4^+^ T cells (Figure 3A and 3B). Regarding absolute T-cell numbers, the CD8+ T-cell density significantly increased while the CD4^+^ T-cell levels were mildly elevated in mice receiving single anti-CD137 mAb (Figure 3I and 3J). In contrast to anti-CD137, blockade of TIM-3 increased the percentage of CD4^+^ and CD8^+^ effector T-cells infiltrating the tumors with former more pronounced (Figure 3A and 3B). Combined treatment with both mAbs benefits from the increased CD8^+^ and CD4^+^ effector T-cell infiltration elicited by anti-TIM-3 and anti-CD137 respectively, resulting in the much higher level of these effector T cells in TIIC compared with that from control treated mice (Figure 3A and 3B).

We also assessed the effects of combined treatment on Treg and MDSC using CD4^+^FoxP3^+^ and CD11b^+^GR-1^+^ makers respectively. While both mAbs moderately decreased the fraction of Treg and MDSC in TIIC (Figure 3C and 3D), CD137 activation increased absolute numbers of Treg and MDSC, whereas TIM-3 blockade did not (Figure 3K and 3L). Thus, combined anti-TIM-3/CD137 treatment not only further diminish the percentages of Treg and MDSC in TIIC compared with either mAb alone, but also reduce the numbers of Treg and MDSC to those achieved with anti-TIM-3 alone.

By increasing CD8 infiltration and attenuating accumulation of Treg and MDSC, combined anti-TIM-3/CD137 treatment strongly increased intra-tumoral CD8 to Treg and MDSC ratios (Figure 3E and 3F). The ratios of CD4 effector to Treg and MDSC in the peritoneal lavage were also elevated in combined treatment, largely due to the effects of TIM-3 blockade in promoting increased CD4 infiltration (Figure 3G and 3H). We conclude that the combined treatment creates higher CD8/Treg (23.75 versus 4.062, p < 0.01) and CD8/MDSC (8.911 versus 1.622, p < 0.01) ratios than TIM-3 blockade alone, while also providing a significantly higher CD4/Treg (23.05 versus 5.860, p < 0.05) and CD4/MDSC (8.672 versus 2.268, p < 0.05) ratio compared to anti-CD137 alone. This combination of both high CD8 and CD4 effector to Treg and MDSC ratios in peritoneal cavity represents the shift of the normally suppressive tumor melieu to a more stimulatory state which is more permissive for immune mediated tumor destruction.

### Combined anti-TIM-3/CD137 mAb treatment shifted a marked gene expression signature typical of Th1 immune response

**Figure 4:**
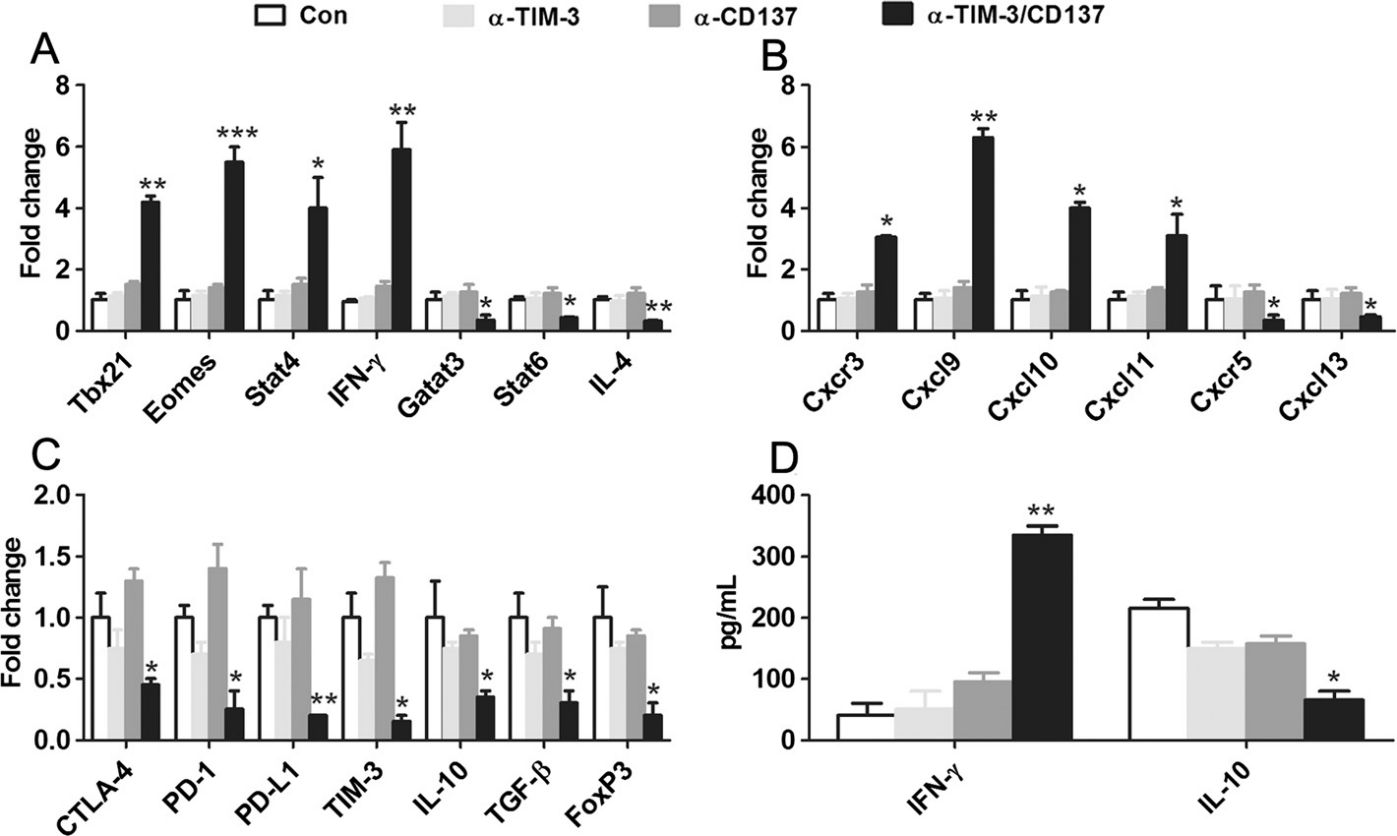
**Immune-related gene expression in peritoneal lavage from treated and control mice with ID8 tumor. A**, Quantitative RT-PCR data demonstrating upregulated transcription of the Th1-related molecules Tbx21, Eomes, IFN-γ and Stat4 and downregulated transcription of the Th2 molecules IL-4, Stat6, and GATA3 in 2 mAb-treated mice. **B**, Quantitative RT-PCR data demonstrating elevation of the Th1-associated chemokine/receptor CXCL9-11/CXCR3 and decrease of the B-cell chemotactic chemokine/receptor CXCL13/CXCR5 in cells of peritoneal lavage from 2 mAb-treated mice. **C**, Results from quantitative RT-PCR demonstrating that mRNA for CTLA-4, PD-1, PD-L1, TIM-3, IL-10, TGF-β and FoxP3 was significantly decreased in cells of peritoneal lavage from 2 mAb-treated mice. **D**, IL-2 and IFN-γ production in the supernatants from pooled lavage cells harvested from treated mice which were stimulated in vitro with 50 ng/ml PMA and 1 μg/ml ionomycin for 6 hours prior to analysis. *P < 0.05, **P < 0.01, ***P < 0.001, compared with control mAb- or single mAb-treated mice.

To further study the local immune effectors induced by anti-TIM-3/CD137 treatment, we performed quantitative RT-PCR to compare the transcription profile of 20 immune gene products expressed in cells from peritoneal lavage, which were collected 7 days after the third injection of mAb, with that of control mAb-treated mice. As shown in Figure 4A, the Th1 transcription factors Stat4, Eomes and Tbx21 (also called T-bet) and their target, IFN-γ, were increased by 4–6 fold in anti-TIM-3/CD137 versus control-treated lavage cells, in contrast, the 2 mAb combination downreguled transcription of the Th2 molecules IL-4, Stat6, and GATA3 (Figure 4B). There was also an increase of the Th1-associated chemokine/receptor CXCL9-11/CXCR3 and a decrease of the Th2-associated chemokine/receptor CXCL13/CXCR5 (Figure 4C), which is concordant with the increase of Th1-type CD4^+^ and CD8^+^ T cells in peritoneal lavage (Figure 3A and 3B). In addition, expression of the genes associated with negative immune regulation, including CTLA-4, PD-1, PD-L1, TIM-3, IL-10, TGF-β and FoxP3, significantly decreased in lavage cells from 2 mAb treated mice compared with that from control- or single mAb-treated mice (Figure 4C); Treatment with single mAb, in particular single anti-CD137 mAb, moderately increased the expression of both Th1- and Th2-associated genes in lavage cells. The contrasting effect was seen on the expression of CTLA-4, PD-1, PD-L1 and TIM-3 co-inhibitory molecules with anti-CD137 mAb upregulated and anti-TIM-3 mAb downregulated the expression of these molecules respectively. Both of them decreased the expression of IL-10, TGF-β and FoxP3 genes in lavage cells. All changes between single mAb and control mAb did not reach statistical significance. Consistent with the data from quantitative RT-PCR, lavage cells from 2 mAb combination produced significantly higher level of IFN-γ while decreased level of IL-10 compared with that from control or single mAb upon polyclonal stimulation (Figure 4D). The data support a shift of the immune response toward immune-stimulatory Th1 type by the 2 mAb treatment.

### Combined anti-TIM-3/CD137 mAb treatment elicited an antigen-specific CTL response

**Figure 5:**
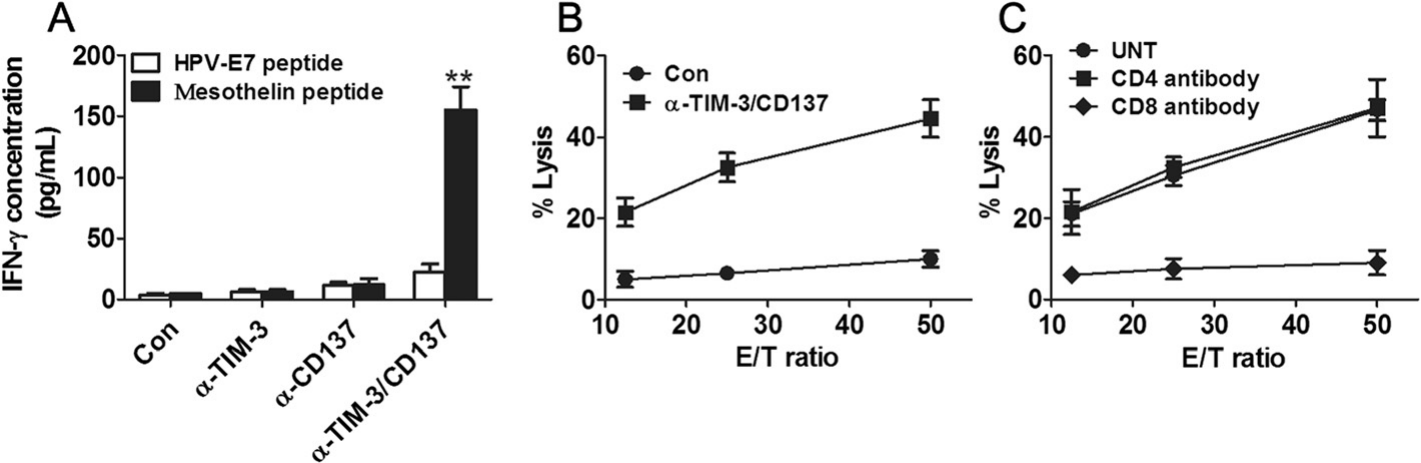
**Mice injected with anti-TIM-3/CD137 mAb developed a tumor antigen-specific CTL response. A**, mice injected i.p. with 1 × 10^6^ ID8 cells 10 day earlier were injected thrice at 4 days interval with 250 μg of anti-TIM-3/CD137 mAb. Seven days after the last mAb injection, splenocytes from treated mice were cultured in the presence or absence of H-2Db-restricted mesothelin-derived peptides or control HPV-E7-derived peptide for 3 days and IFN-γ production in the supernatants were assayed by ELISA. **B**, mice were treated as described in **(A)**. Seven days after the last mAb injection, splenocytes (5 × 10^6^) from 3 mice were incubated with 5 × 10^5^ UV-irradiated ID8 cells for 4 days prior to subject to analysis of antigen-specific CTL activity by CytoTox 96 Non-radioactive cytotoxicity assay using EL4 cells pulsed with H-2Db-restricted mesothelin or HPV-E7 peptide as target cells. The killing activity was also evaluated in the presence of anti-CD4, anti-CD8 or control antibody **(C)**. Data were expressed as M ± SEM of triplicate wells.

As ID8 cells express the mesothelin (Additional file 3: Figure S2), a well-characterized tumor antigen [8], we harvested splenocytes from 2 mAb and control mAb treated mice, and the same number of splenocytes was cultured in the presence of 10 μg/mL of H-2Db-restricted mesothelin-specific peptide (amino acid 406–414) or control HPV-E7 peptide (amino acids 49–57) for 3 days and assayed the culture supernatants for IFN-γ by ELISA. Splenocytes from 2 mAb-treated mice, as compared with control or single mAb-treated mice, secreted significantly elevated levels of IFN-γ when stimulated with the mesothelin peptide as compared with stimulation by the HPV peptide (P < 0.01; Figure 5A). We further determined whether the splenocytes had cytotoxic activity. Splenocytes from mice treated with anti-TIM-3/CD137 mAbs were restimulated with UV-irradiated ID8 cells for 4 days before CTL assays were performed using EL4 cells pulsed with mesothelin or HPV-E7 derived peptide as target cells. As shown in Figure 5B, splenocytes from anti-TIM-3/CD137 treated mice displayed cytotoxic activity on EL4 cell pulsed with mesothelin but not with HPV-E7 peptide. Pre-incubation with CD8 antibody suppressed the killing activity (Figure 5C) and we conclude that combined anti-TIM-3/CD137 mAbs elicited a tumor antigen-specific CTL response mediated by CD8^+^ T cells. We also evaluated the presence of mesothelin-specific antibodies in sera from the 2 mAb treated long-term surviving mice by flow cytometry using mesothelin-expressing ID8 cells as detecting cells. Compared with sera from naïve mice, we did not see a clear increase of fluorescence intensity from sera from those long-term survivors (data not shown), suggesting the absence of mesothelin-specific antibodies in these mice.

## Discussion

The antitumor efficacy of immunotherapy remains insufficient to achieve durable clinical responses in patients with advanced EOC. In this study, we demonstrate that combined anti-TIM-3/CD137 mAbs inhibited the outgrowth of ID8 ovarian cancer cells injected 10 days previously, resulting in the long-lasting survival of 60% of mice while either mAb alone was ineffective in tumor protection. The findings provide evidence that combined TIM-3 blockade and CD137 activation may serve as a novel immunotherapeutic option for treatment of ovarian cancer.

We next sought to understand the mechanisms underlying increased tumor-rejecting effect by simultaneously removing a major brake on expansion via blockade of the negative regulator TIM-3, while at the same time actively driving proliferation and survival through activation of the co-stimulatory receptor CD137. We found that single TIM-3 blockade significantly increased the percentage of CD4^+^ T cells and slightly elevated the percentage of CD8^+^ T cells while it had little effects on the immunosuppressive CD4^+^FoxP3^+^ Treg, CD11b^+^GR-1^+^ MDSCs and CD19^+^ B cells in peritoneal lavage. On the contrast, single CD137 activation promoted the accumulation of CD8^+^ T cells with significantly elevated percentage and absolute number in peritoneal lavage. Quantitative RT-PCR data demonstrated moderately increased IFN-γ expression although significantly increased accumulation of CD8^+^ T cells in peritoneal cavity from anti-CD137 mAb treated mice, indicating most of CD8^+^ T cells were without function or immunologically ignorant as described previously [30]. This was consistent with the lack of antitumor effect by single anti-CD137 mAb in ID8 model, which may be due to the concomitant expansion of Treg and MDSC by anti-CD137 mAb. In accordance with the more pronounced CD137 expression on CD8^+^ versus CD4^+^ T cells, marginal effect of anti-CD137 mAb on CD4^+^ T cells were observed. Remarkably, combined anti-TIM-3/CD137 mAb increased the percentage and absolute number of tumor-infiltrating CD4^+^ and CD8^+^ T cells, while at the same time decreasing the immunosuppressive Treg and MDSC in peritoneal lavage, which gave rise to the significantly increased ratio of CD4^+^ and CD8^+^ T cells to immunosuppressive cells.

Importantly, we detected a systemic antigen-specific CD8^+^ T-cell mediated CTL immune response to mouse mesothelin in anti-TIM-3/CD137 mAb treated mice with ID8 tumors, as evidenced by mesothelin epitope-specific IFN-γ production and cytotoxicity by CD8^+^ T cells from these mice. As an endogenous non-mutated antigen, mesothelin should be naturally tolerized against. The induction of mesothelin-specific CD8^+^ CTL by anti-TIM-3/CD137 mAb in the mesothelin-expressing ID8 model indicates that endogenous tolerance to mesothelin was overcome, which is consistent with previous studies showing the presence of mesothelin-specific humoral or cellular immune response in patients with cancer expressing high level of mesothelin, such as pancreatic cancer, ovarian cancer or mesothelioma [31,32]. We did not detect mesothelin-specific antibodies in sera harvested 90 days after tumor challenge in 2 mAb treated mice by flow cytometry, but the presence of these antibodies cannot be completely excluded in view of comparatively low sensitivity of this approach and possibly suboptimal time point for sera collection. Serially collection of sera at different time points after mAb injection and utilization of more sensitive approaches such as ELISA should be warrant in our future work.

The importance of CD8^+^ T-cell mediated CTL response in tumor protection was supported by in vivo antibody depletion experiments demonstrating depletion of CD8^+^ T cells abrogated the antitumor effect of anti-TIM-3/CD137 mAb. The pivotal role of CD8^+^ T cells in antitumor effect elicited by anti-TIM-3/CD137 mAb treatment is consistent with other combined strategies involving anti-CD137 mAb, such as combined cyclophosphamide/anti-CD137 treatment [33], which has been shown to produce synergist antitumor effects via expansion of CD8^+^ tumor-specific T cells. The depletion of CD4^+^ T cells also decreased the antitumor effect of 2 mAb treatment although the effect was not as prominent as depletion of CD8^+^ T cells, which may be explained by the fact that Treg depletion by CD4 antibody partially compensates a deleterious effect on effector CD4^+^ T cells in view of the finding that Treg cells can be expanded by anti-CD137 antibody [34]. Our data were concordant with a recent study showing the administration of anti-TIM-3 mAb had a tumor-suppressing effect in several transplantable and chemical carcinogen-induced fibrosarcoma tumor models via CD4^+^ and CD8^+^ T-cell- and IFN-γ-dependent mechanisms [16,35]. A recent study also shows that TIM-3 blockade enhanced the antitumor effects of vaccine-induced response against established B16 murine melanomas via NK cell-dependent mechanisms [36], and this discrepancy may be possibly due to the different treatments and tumor microenvironments where TIM-3 may modulate distinct immune cells and the related signaling pathways that exist [36].

Noticeably, we did not detect the expression of galectin 9 on the tumor cells in spite of the immune enhancing effect of the anti-TIM3 mAb. This finding is contrasting to the scenario of other inhibitory receptors, such as PD-1, where the presence of the ligand (PD-L1) on the tumor seems to correlate with the response to anti-PD-1 [37]. A recent report did not detect a specific interaction between galectin 9 and TIM-3 [38], suggesting that TIM-3 functions are independent of galectin-9, which may partially explain our finding. It warrants further explore whether TIM-3 receives an inhibitory signal from an unidentified molecule other than galectin-9 on tumor cells where blockade of this interaction by anti-TIM-3 mAb elicits an immunostimulatory effect.

Activation of the co-stimulatory receptor CD137 in the clinic has shown promise as a therapy for advanced solid tumors with manageable autoimmune adverse effects when administrated at dose levels ranging from 0.3 mg per kg to 10 mg per kg [27]. The major side effect of systemic use of CD137 agonist antibody appears to be an as yet poorly mechanistically defined inflammatory liver toxicity by infiltration with T cells and hematologic abnormalities [39,40]. In our study, we did not observe any obvious toxicity such as weight or hair loss in mice receiving single or combined anti-TIM-3/CD137 mAb. Further detailed biochemical and histological analysis of liver, spleen, bone marrow and peripheral blood at different time points after antibody injection should be warranted to inform any major side effects induced by 2 mAb treatment. Recent studies provide a rationale for local delivery of anti-CD137 mAb to treat tumor. The study from A. Palazon et al. shows that hypoxia-inducing transcription factor-1α (HIF-1α) in hypoxic tumors induces the expression of CD137 on TILs [41] and local intratumoral low-dose injection of agonist anti-CD137 mAb elicited systemic tumor-specific effector T cells capable of eradicating distant metastases. In addition, a study performed in a murine AT3 breast cancer model shows that combined radiotherapy and anti-CD137 treatment upregulated the expression of CD137 on tumor-specific CD8^+^ CTLs [42]. Therefore, it is of great interest to elucidate the role of CD137 expression on TILs in our model since local delivery of anti-CD137 antibodies might avoid the toxicity associated with systemic application without compromising the antitumor effects.

In view of recent encouraging results of immunomodulatory mAbs in clinic for treatment of multiple solid tumor [43], our finding that TIM-3 blockade and CD137 activation synergistically induce a potent antitumor effect in a highly clinical relevant ID8 ovarian cancer model should aid the design of future trials for ovarian cancer immunotherapy.

## Abbreviations

TIM-3: T cell immunoglobulin and mucin domain 3; mAb: Monoclonal antibody; TNFR: Tumor necrosis factor receptor; Th1: T-helper 1; Tc1: T cytotoxic type 1; CTL: Cytotoxicity T lymphocytes; Treg: Regulatory T cells; MDSC: Myeloid-derived suppressor cells; EOC: Epithelial ovarian carcinoma; IFN-γ: Interferon-gamma; PD-1: Programmed death protein 1; TNFR: Tumor necrosis factor receptor; IL: Interleukin; mAb: Monoclonal antibody; DMEM: Dulbecco’s minimum essential medium; RPMI: Roswell park memorial institute; NK: Natural killer; DC: Dendritic cells; PBS: Phosphate-buffered saline; FBS: Fetal bovine serum; RT-PCR: Reverse transcription polymerase chain reaction; GAPDH: Glyceraldehyde phosphate dehydrogenase; SEM: Standard error of mean.

## Competing interests

The authors declare that they have no competing interests.

## Authors’ contributions

ZQG conceived and designed the study, performed most of the experiments and drafted the manuscript. LLW and GW carried out the flow cytometric analysis, participated in the design of the study and helped in writing the manuscript. DLC contributed in cell culture techniques and analyzed data. ZJX participated in the statistical analysis and interpretation of data. ML participated in the analysis and revised the manuscript. SLZ, head of the department, critically revised the manuscript. All authors read and approved the final manuscript.

## Supplementary Material

Additional file 1: Figure S1 The typical presentation of ID8 ovarian cancer in C57BL/6 mice. The left and right picture shows the macroscopic appearance of ascites and ID8 tumor mass in peritoneal cavity of mice respectively. Click here for file

Additional file 2: Table S1 Primers used in Real-Time PCR. Click here for file

Additional file 3: Figure S2 The expression of mesothelin antigen in ID8 cells. For detection of mesothelin expression on cell surface (left histogram), ID8 cells was directly stained with PE labeled anti-mesothelin (red line) or isotype-matched mAb (black line); for detection of intracellular mesothelin, ID8 cells were fixed and permeabilized using Cytofix/Cytoperm kit prior to staining with antibodies as above. The mesothelin expression was analyzed by flow cytometry. All antibodies were purchased from R&D system. Click here for file
