# Supplementary figures and images for: Combined TIM-3 blockade and CD137 activation affords the long-term protection in a murine model of ovarian cancer
Source: J Transl Med. 2013 Sep 17;11:215. doi: 10.1186/1479-5876-11-215 (PMC3853027; doi:10.1186/1479-5876-11-215)

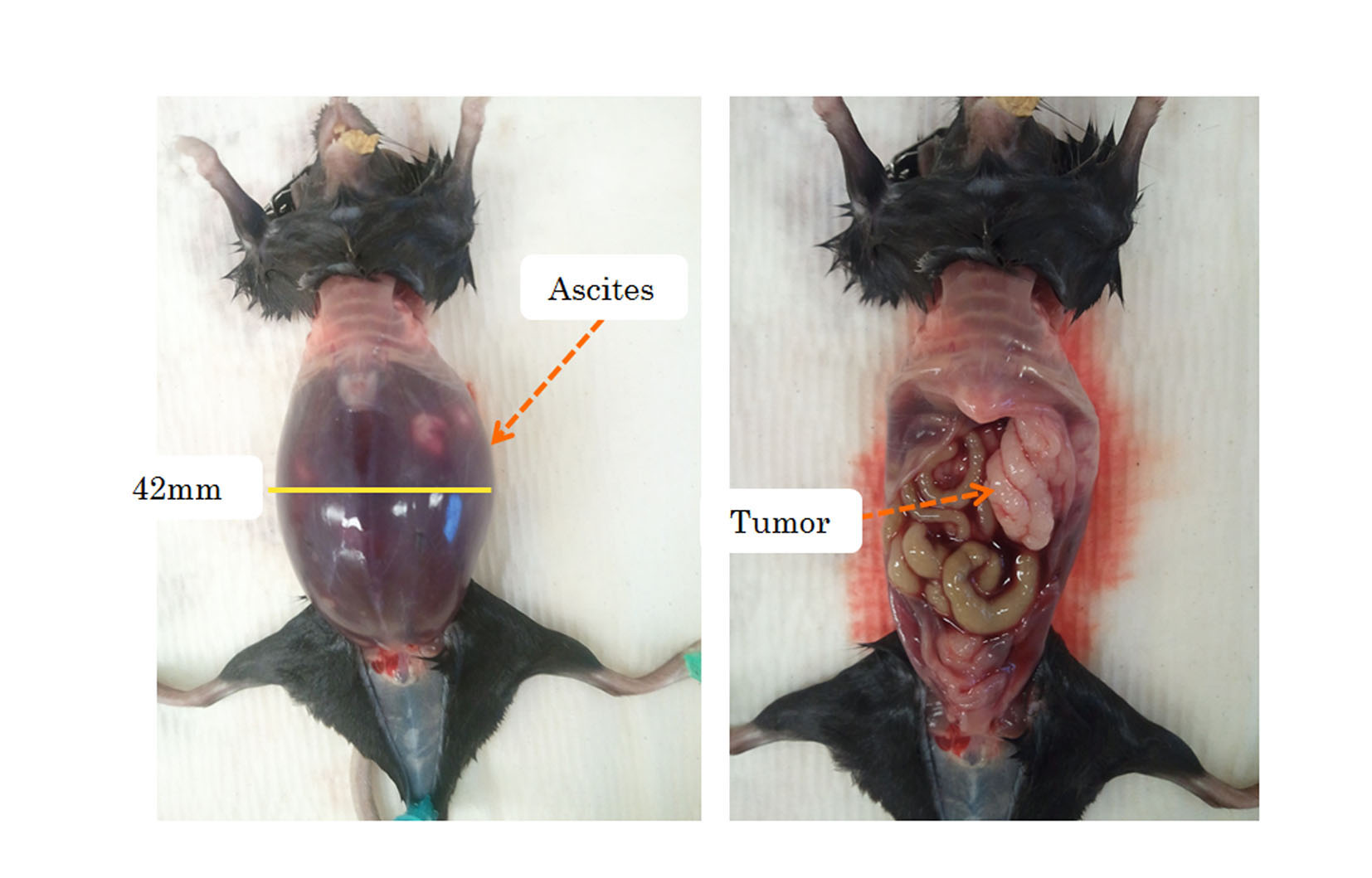

Supplement: Additional file 1: Figure S1 — The typical presentation of ID8 ovarian cancer in C57BL/6 mice. The left and right picture shows the macroscopic appearance of ascites and ID8 tumor mass in peritoneal cavity of mice respectively. [file 1479-5876-11-215-S1.tiff]

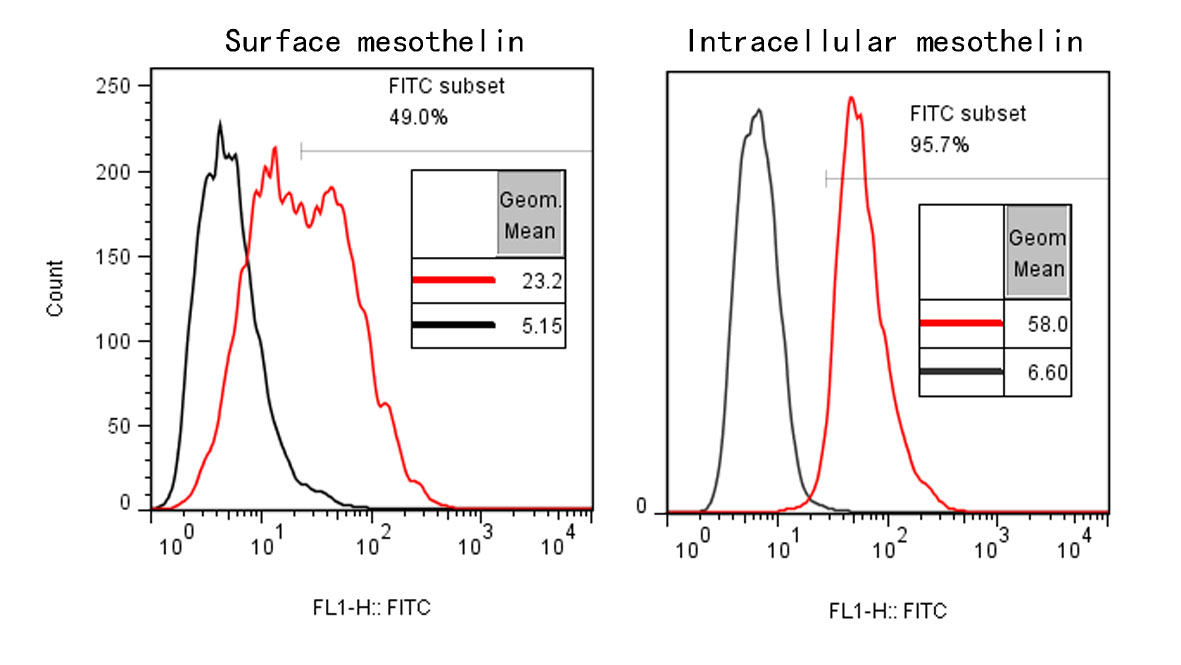

Supplement: Additional file 3: Figure S2 — The expression of mesothelin antigen in ID8 cells. For detection of mesothelin expression on cell surface (left histogram), ID8 cells was directly stained with PE labeled anti-mesothelin (red line) or isotype-matched mAb (black line); for detection of intracellular mesothelin, ID8 cells were fixed and permeabilized using Cytofix/Cytoperm kit prior to staining with antibodies as above. The mesothelin expression was analyzed by flow cytometry. All antibodies were purchased from R&D system. [file 1479-5876-11-215-S3.tiff]
