# Supplementary material for: Combined TIM-3 blockade and CD137 activation affords the long-term protection in a murine model of ovarian cancer
Source: J Transl Med. 2013 Sep 17;11:215. doi: 10.1186/1479-5876-11-215 (PMC3853027; doi:10.1186/1479-5876-11-215)
Supplement: Additional file 2: Table S1 — Primers used in Real-Time PCR. [file 1479-5876-11-215-S2.docx]

**Table S1. Primers used in Real-Time PCR**

| GAPDH | Sense:5′-GTGGAGATTGTTGCCATCAACG-3′  Antisense:5′-CAGTGGATGCAGGGATGATGTTCTG-3′ |
| --- | --- |
| TGF-β1 | Sense:5′-GTGGTATACTGAGACACCTTGG-3′  Antisense:5′-CCTTAGTTTGGACAGGATCTGG-3′ |
| IL-10 | Sense:5′-CTCTTACTGACTGGCATGAGG-3′  Antisense:5′-CCTTGTAGACACCTTGGTCTTGGAG-3′ |
| FoxP3 | Sense:5′-CAGCTGCCTACAGTGCCCCTAG-3′  Antisense:5′-CATTTGCCAGCAGTGGGTAG-3′ |
| PD-1 | Sense: 5′-TTC AGG TTT ACC ACA AGC TGG-3′  Antisense: 5′-TGA CAA TAG GAA ACC GGG AA-3′ |
| PD-L1 | Sense: 5′-GGAATTGTCTCAGAATGGTC-3′  Antisense: 5′-GTAGTTGCTTCTAGGAAGGAG-3′ |
| TIM-3 | Sense: 5′-GACCCTCCATAATAACAA-3′  Antisense: 5′-TAATAAGGCTCAAACTCG-3′ |
| CTLA-4 | Sense: 5′-CTACCTGGGCATAGGCAACG-3′  Antisense: 5′-CCCCGAACTAACTGCTGCAA-3′ |
| IFN-γ | Sense: 5′-AAAAACCTAAAAAATCTAAATAACT-3′  Antisense: 5′-ATCAACAACAACTCCTTTTCCACTT-3′ |
| TBX21 | Sense: 5′-CGGTACCAGAGCGGCAAGT-3′  Antisense: 5′-AGCCCCCTTGTTGTTGGTG-3′ |
| GATA-3 | Sense: 5′-TCTCACTCTCGAGGCAGCATGA-3′  Antisense: 5′-GGTACCATCTCGCCGCCACAG-3′ |
| IL-4 | Sense:5′-GGTCTCAACCCCCAGCTAGT-3′  Antisense: 5′-GCCGATGATCTCTCTCAAGTGAT-3′ |
| STAT6 | Sense:5′-AGTGCAGCGGCTCTATGTCGAC-3′  Antisense: 5′-CACCGAGGCCTGAAGGTGC-3′ |
| STAT4 | Sense:5′-CCTGACATTCCCAAAGACAAAGC-3′  Antisense: 5′-TCTCTCAGCACAGCATATGCAC-3′ |
| CXCL9 | Sense:5′-TTTTGGGCATCATCTTCCTGG-3′  Antisense: 5′-GAGGTCTTTGAGGGATTTGTAGTGG-3′ |
| CXCL10 | Sense:5′-CTTCTGAAAGGTGACCAGCC-3′  Antisense: 5′-GTCGCACCTCCACATAGCTT-3′ |
| CXCL11 | Sense:5′-AACAGGAAGGTCACAGCCATAGC-3′  Antisense: 5′-TTTGTCGCAGCCGTTACTCG-3′ |
| CXCR3 | Sense:5′-CTGCTGCCCAGTGGGTTT-3′  Antisense: 5′-GTTGATGTTGAACAGGGCACC-3′ |
| CXCL13 | Sense:5′-gaggcagatgga act tgagc-3′  Antisense: 5′-ctggggatcttcgaatgcta-3′ |
| CXCR5 | Sense:5′-tacccgctaacgctgaaatggac-3′  Antisense: 5′-cacggcaaagggcaagatgaagac-3′ |
